# Prognostic value of a left atrioventricular coupling index in pre- and post-menopausal women from the Multi-Ethnic Study of Atherosclerosis

**DOI:** 10.3389/fcvm.2022.1066849

**Published:** 2022-11-21

**Authors:** Théo Pezel, Erin D. Michos, Vinithra Varadarajan, Mahsima Shabani, Bharath Ambale Venkatesh, Dhananjay Vaidya, Yoko Kato, Henrique Doria De Vasconcellos, Susan R. Heckbert, Colin O. Wu, Wendy S. Post, David A. Bluemke, Matthew A. Allison, Patrick Henry, Joao A. C. Lima

**Affiliations:** ^1^Division of Cardiology, Johns Hopkins University, Baltimore, MD, United States; ^2^Université de Paris Cité, Service de Cardiologie, Hôpital Universitaire Lariboisière – APHP, Paris, France; ^3^Department of Medicine Division of General Medicine, The Johns Hopkins University, Baltimore, MD, United States; ^4^Department of Epidemiology, University of Washington, Seattle, WA, United States; ^5^Division of Intramural Research, National Heart Lung and Blood Institute, Bethesda, MD, United States; ^6^Department of Radiology, University of Wisconsin School of Medicine and Public Health, Madison, WI, United States; ^7^Division of Preventive Medicine, Department of Family Medicine and Public Health, University of California, San Diego, La Jolla, CA, United States

**Keywords:** Multi-Ethnic Study of Atherosclerosis (MESA), menopause, left atrioventricular coupling, cardiovascular magnetic resonanace, prognosis, sex hormones (SH), cardiovascular events (CVE)

## Abstract

**Background:**

Sex hormones associated with both the left atrial (LA) and left ventricular (LV) structures in women, but the association of menopause status with left atrioventricular coupling is not established.

**Aim:**

To assess the prognostic value of a left atrioventricular coupling index (LACI) in peri-menopausal women without a history of cardiovascular disease (CVD).

**Materials and methods:**

In all women participating in MESA study with baseline cardiovascular MRI, the LACI was measured as the ratio of the LA end-diastolic volume to the LV end-diastolic volume. Cox models were used to assess the association between the LACI and the outcomes of atrial fibrillation (AF), heart failure (HF), coronary heart disease (CHD) death, and hard CVD.

**Results:**

Among the 2,087 women participants (61 ± 10 years), 485 cardiovascular events occurred (mean follow-up: 13.2 ± 3.3 years). A higher LACI was independently associated with AF (HR 1.70; 95%CI [1.51–1.90]), HF (HR 1.62; [1.33–1.97]), CHD death (HR 1.36; [1.10–1.68]), and hard CVD (HR 1.30; [1.13–1.51], all *p* < 0.001). Adjusted models with the LACI showed significant improvement in model discrimination and reclassification when compared to traditional models to predict: incident AF (C-statistic: 0.82 vs. 0.79; NRI = 0.325; IDI = 0.036), HF (C-statistic: 0.84 vs. 0.81; NRI = 0.571; IDI = 0.023), CHD death (C-statistic: 0.87 vs. 0.85; NRI = 0.506; IDI = 0.012), hard CVD (C-statistic: 0.78 vs. 0.76; NRI = 0.229; IDI = 0.012). The prognostic value of the LACI had a better discrimination and reclassification than individual LA or LV parameters.

**Conclusion:**

In a multi-ethnic population of pre- and post-menopausal women, the LACI is an independent predictor of HF, AF, CHD death, and hard CVD.

**Clinical trial registration:**

[https://clinicaltrials.gov/], identifier [NCT00005487].

## Introduction

Although the risk of cardiovascular disease (CVD) is much lower in women than in men until 50 years of age, it rises dramatically after menopause (1, 2). This higher rate of cardiovascular events in peri- and post-menopausal women contributes significantly to morbi-mortality rates among women worldwide (3, 4). It has been hypothesized that the increased CVD risk in peri-menopausal women is due to lower levels of endogenous estrogens and higher levels of endogenous androgens during and after the menopausal transition (1, 5). In line with this hypothesis, a study from the Multi-Ethnic Study of Atherosclerosis (MESA), which assessed 2,834 post-menopausal women, recently showed that a more androgenic sex hormone profile was associated with women’s increased CVD risk later in life, including coronary heart disease (CHD), and incident heart failure (HF) (6). Another study recently provided evidence of a significant relationship between post-menopausal hormone levels and changes in left ventricular (LV) structure (7).

The protective role of estrogen on the cardiovascular system has already been described, along with the mediating mechanisms, such as fibrosis, oxidative stress, vascular function, and ventricular remodeling (8). Regarding this last point, a very recent study described the modifications caused by LV remodeling due to menopause using cardiovascular magnetic resonance (CMR) in 14,550 post-menopausal women (9). In this study, menopause was independently associated with smaller LV end-diastolic volume and smaller LV stroke volume (9). Beyond these effects on LV remodeling, some studies have also suggested a potential repercussion of menopause on left atrial (LA) remodeling (10, 11). Therefore, we can assume that hormone levels could cause LA remodeling and functional change in peri- and post-menopausal women.

Given that sex hormone levels may cause both LA and LV remodeling, it is relevant to assess the potential consequences of the menopausal transition on left atrioventricular coupling. Indeed, the left atrium and ventricle interact throughout the cardiac cycle. During diastole, the LA and LV are directly connected, and in the absence of valvular disease, their function is tightly coupled (12). However, there is a paucity of literature on the ability of atrioventricular coupled indexes to predict outcomes with very limited sample sizes (13, 14). Recently, our working group demonstrated the prognostic value of a novel left atrioventricular coupling index (LACI) defined by the ratio of the LA end-diastolic volume divided by the LV end-diastolic volume using CMR (15). Those prior MESA results, conducted in both men and women, suggested that an increase in LACI is associated with cardiovascular outcomes. Using a longitudinal analysis, our team has also shown the incremental prognostic value of annualized change in LACI over 10 years (?LACI), along with traditional risk factors, which had a better prognostic value than individual changes in LA or LV parameters measured separately to predict incident HF (16), and incident atrial fibrillation (AF) (17). More recently, Meucci et al. showed its strong prognostic value for incident AF in patients with hypertrophic cardiomyopathy (18). All these findings support the physiological concept of left atrioventricular coupling by this LA/LV coupling index, which has a better prognostic value than individual LA or LV parameters measured separately (19). Despite peri- and post-menopausal women presenting a higher rate of cardiovascular events (1, 5), the potential role of the menopause transition on left atrioventricular coupling in this population is not well established.

Therefore, we hypothesized that the menopausal transition plays a potential role through endogenous sex hormones in left atrioventricular coupling. Hence, we investigated the relationship between the LACI and the menopausal status and sex hormone levels, and then evaluated the prognostic value of the LACI to predict the occurrence of cardiovascular events in pre- and post-menopausal women in the MESA.

## Materials and methods

### Study population

The MESA is a prospective, community-based, multi-ethnic (consisting of self-reported White, African American, Chinese, and Hispanic racia/ethnic groups) cohort study evaluating subclinical CVD and its progression to clinical CVD. The study design of the MESA has been described in detail previously (20). In summary, between 2000 and 2002 (Exam 1), 6,814 men and women aged 45–84 years and free of clinical CVD at enrollment were consecutively recruited from six US field centers (Baltimore, MD; Chicago, IL; Forsyth County, NC; Los Angeles County, CA; Northern Manhattan, NY; and St Paul, MN). Participants with clinically significant CVD at baseline were excluded.

The current analysis included all women assessed by CMR at the baseline. Menopausal status was self-reported and corrected using information on age, self-reported hysterectomy, bilateral oophorectomy, and menopausal age. Baseline serum sex hormones were measured following a MESA protocol already described (7) and included estradiol, free and total testosterone (T), dehydroepiandrosterone (DHEA), and sex hormone binding globulin (SHBG). Total testosterone was divided by estradiol to calculate the total testosterone-to-estradiol ratio. The methodology of baseline characteristics collection is detailed in Supplementary material 1. All participants provided written informed consent. All study protocols were approved by the institutional review boards of each participating field center.

**FIGURE 1:**
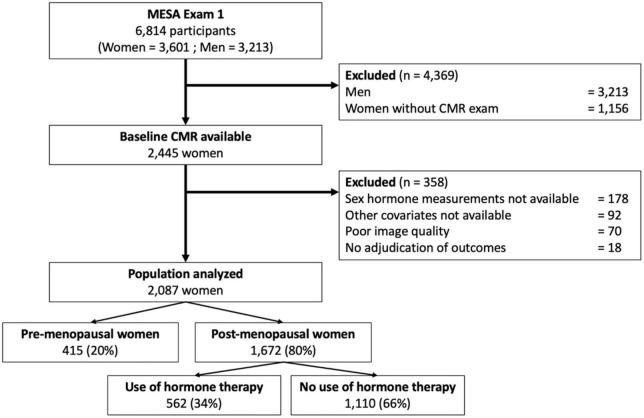
Flowchart of the study.

A flowchart of the MESA population investigated in this study is depicted in Figure 1.

Of the 2,248 women with CMR imaging data available that included LA volume assessment (Exam 1), 21 individuals had no follow-up for cardiovascular events, 77 had missing images or insufficient image quality to measure LA and LV volumes, and a further 63 had missing covariates. This resulted in a final cohort of 2,087 women available for analysis (Figure 1).

### Cardiovascular magnetic resonance protocol and image analysis

The CMR protocol has been described in detail previously (21). Briefly, CMR was performed at six MESA field centers using 1.5-Tesla scanners (Supplementary material 2). Long-axis cine images were obtained from two- and four-chamber views using electrocardiogram-gated fast gradient-echo pulse sequences. A stack of short-axis cine images was acquired to encompass both ventricles, and LV end-diastolic volume was measured using cardiac image modeler software (CIM version-6.0, Auckland University, New Zealand). All cine images were acquired with a temporal resolution of ∼50 ms. The complete CMR protocol as well as details of image analysis, data quality control, calculations for left ventricular ejection fraction (LVEF), LV mass and volumes, LA volumes, and reproducibility of these measurements have been published previously (21).

Multimodality tissue tracking software (MTT version-6.0, Toshiba Medical, Tokyo, Japan) was used to quantify LA volume and strain from two- and four- chamber cine CMR images (Supplementary material 3). This method has been validated previously, showing good to excellent intra- and inter-reader reproducibility, with intraclass correlation coefficient (ICC) of 0.88–0.98 (*p* < 0.001), and reasonable inter-study reproducibility, with ICC of 0.44–0.82 (*p* < 0.05–0.001) (22–24). A single experienced operator blinded to the case status of the participant defined endocardial and epicardial borders of the LA at end-diastole. Using the marked points, the software created endocardial and epicardial borders, and then tracked LA tissue in subsequent frames.

### Left atrioventricular coupling index

As recently described by our group (15), the LACI was defined as the ratio of the LA end-diastolic volume to the LV end-diastolic volume, as assessed using CMR. The LV volume was measured from the stack of short-axis cine images, while the LA volume was measured from the two- and four-chamber views, as previously described. The LA and LV volumes were measured with a match in the same end-diastolic phase defined by mitral valve closure. The intra- and inter-reader reproducibility of the LACI were both good, with ICC of 0.93 (95% confidence interval, CI 0.90–0.96) and 0.81 (95% CI 0.71–0.88), respectively (15). The LACI value is expressed as a percentage, and a higher LACI expresses greater disproportion between the LA and LV volumes at ventricular end diastole, reflecting greater impairment of left atrioventricular coupling.

### Outcomes

The MESA outcome ascertainment protocols have been described in detail and are available online.^1^ Outcomes of interest were HF, AF, CHD death, and hard CVD (Supplementary material 4). In addition to MESA follow-up examinations every two years, a telephone interviewer contacted each participant every 9–12 months to inquire about interim hospital admissions, cardiovascular outpatient diagnoses, and death. Two physicians reviewed all records for independent endpoint classification and the assignment of event dates. Criteria for hard CVD outcomes included myocardial infarction, resuscitated cardiac arrest, death from coronary disease, and fatal and non-fatal stroke. CHD death included myocardial infarction, chest pain within the 72 hours before death, or a history of CHD and the absence of a non-cardiac cause of death. Criteria for HF as an endpoint included symptomatic HF diagnosed by a physician in a patient receiving medical treatment for HF and exhibiting pulmonary edema/congestion by chest X-ray and/or a dilated LV or poor LV function by echocardiography, or evidence of LV diastolic dysfunction. Criteria for AF as an endpoint required an AF diagnosis according to International Classification of Diseases (ICD)-9 codes.

### Statistical analysis

The baseline characteristics of the study participants are presented as mean ± standard deviation (SD) or median (interquartile range [IQR]) for continuous variables and as counts and percentages for categorical variables in Table 1. The sex hormone levels were positively skewed and thus logarithmically transformed and analyzed per one SD of their natural log. Comparisons employed the χ^2^ or Fisher’s exact test for categorical variables and the Student’s *t*-test or Mann–Whitney–Wilcoxon test, as appropriate, for continuous variables. Cox regression models were used to study the associations between the LACI and the outcomes. The proportional hazard assumption was visually tested using Schoenfeld residuals. The cumulative risk of outcomes over the follow-up years, stratified by LACI tertiles, was determined using Kaplan–Meier curves censored at the most recent follow-up. Differences across terciles were compared using the log-rank test. The associations between LACI or all other LA and LV parameters and time-to-event were analyzed with multivariable Cox survival analyses, adjusting for traditional risk factors at baseline: age, ethnicity, education level, physical activity, menopausal status, hormone therapy (HT) use, diabetes mellitus, current smoking, systolic blood pressure, antihypertensive therapy, body mass index, high-density lipoprotein cholesterol, total cholesterol, lipid-lowering therapy, total testosterone/estradiol ratio, DHEA level, and glomerular filtration rate. Of note, the total testosterone/estradiol ratio was used as a sex hormone covariate in the final model because it was previously described in MESA as the best prognosticator of cardiovascular events in women (6).

**TABLE 1:** Population characteristics of participants at baseline before occurrence of events by incident event categories (*n* = 2,087).

Parameters	No event (*n* = 1,772)	HF (*n* = 83)	AF (*n* = 176)	CHD death (*n* = 71)	Hard CVD (*n* = 155)
Age, years	53.6 ± 10.0	**68.1 ± 8.6**	**69.4 ± 8.1**	**70.0 ± 8.2**	**66.0 ± 9.1**
Ethnicity (Ca/Ch/AA/Hi), %	39/13/26/22	**42/11/28/19**	**51/9/21/19**	38/11/31/20	39/10/26/25
**Education**					
<High school	374 (21.1)	23 (27.7)	52 (29.5)	21 (29.2)	43 (27.7)
High school, technical school, or associate degree	888 (50.1)	45 (54.2)	93 (52.8)	38 (53.7)	84 (54.2)
College, graduate or professional school	500 (28.2)	15 (18.1)	30 (17.0)	12 (17.1)	28 (18.1)
Hypertension, *n* (%)	679 (38.3)	**58 (69.9)**	**102 (58.0)**	**44 (62.4)**	**93 (60.0)**
Systolic blood pressure, mmHg	124 ± 22	**133 ± 23**	**130 ± 21**	**134 ± 21**	**131 ± 22**
Diastolic blood pressure, mmHg	69 ± 10	**71 ± 12**	**70.0 ± 11**	**70.0 ± 10**	**72 ± 11**
Body mass index, kg/m^2^	28.0 ± 5.6	**29.6 ± 5.6**	**29.1 ± 5.5**	28.5 ± 5.4	**29.2 ± 6.1**
Diabetes mellitus, *n* (%)	183 (10.3)	**21 (25.3)**	**27 (15.3)**	**17 (24.2)**	**35 (22.6)**
Smoking status, *n* (%)	195 (11.0)	10 (12.0)	16 (9.1)	**12 (16.3)**	**22 (14.2)**
Heart rate, bpm	64 ± 9	**65 ± 11**	64 ± 9	64 ± 9	**65 ± 10**
Total cholesterol, mg/dl	197 ± 34	**200 ± 33**	192 ± 31	**204 ± 39**	**206 ± 35**
HDL cholesterol, mg/dl	58 ± 15	**54 ± 13**	56 ± 16	**53 ± 14**	**53 ± 14**
eGFR*, ml/min/1.73 m^2^	80.2 ± 15.8	**72.2 ± 19.0**	**70.4 ± 17.3**	**72.6 ± 17.2**	**74.7 ± 18.1**
Hypertension medication, *n* (%)	542 (30.6)	**48 (57.8)**	**90 (51.1)**	**39 (55.2)**	**72 (46.5)**
Lipid-lowering medication, *n* (%)	280 (15.8)	**16 (19.3)**	**35 (19.9)**	**20 (28.2)**	**33 (21.3)**
NT-proBNP, pg/ml	79 (51–109)	**192 (102–391)**	**131 (89–289)**	**120 (69–229)**	**104 (66–298)**
Agatston score, mean ± SD	46 ± 101	**156 ± 316**	**175 ± 431**	**122 ± 310**	**109 ± 212**
Framingham CVD risk,%	9.1 ± 8.7	**17.2 ± 7.3**	**16.8 ± 7.8**	**21.0 ± 7.0**	**17.1 ± 7.8**
CHARGE-AF score	11.5 ± 1.2	**12.8 ± 1.0**	**13.1 ± 1.0**	**12.9 ± 1.0**	**12.3 ± 1.1**
**Baseline LV characteristics**					
LV EDVi, ml/m^2^	66.2 ± 13.0	**71.7 ± 19.0**	**69.5 ± 17.1**	67.5 ± 19.2	66.3 ± 16.0
LVEF,%	63.9 ± 6.1	**60.2 ± 8.0**	63.5 ± 6.6	**61.1 ± 7.8**	**61.8 ± 7.0**
LV mass index, g/m^2^	56.1 ± 12.0	**70.1 ± 17.2**	**65.2 ± 15.1**	**66.3 ± 18.2**	**65.3 ± 15.3**
LVMVR, g/ml	0.88 ± 0.17	**1.01 ± 0.22**	**0.97 ± 0.21**	**1.00 ± 0.22**	**0.99 ± 0.20**
LVGFI,%	43.9 ± 6.2	**36.3 ± 7.0**	39.2 ± 6.3	**37.0 ± 7.2**	**37.3 ± 6.4**
**Baseline LA characteristics**					
LA EDVi, ml/m^2^	11.2 ± 6.1	**16.3 ± 9.8**	**18.6 ± 11.4**	**14.2 ± 9.1**	**13.5 ± 7.6**
LA ESVi, ml/m^2^	29.8 ± 9.9	**35.0 ± 13.6**	**37.5 ± 15.4**	**32.3 ± 13.1**	**31.2 ± 11.6**
Peak LA strain,%	39.8 ± 10.6	**33.1 ± 11.1**	**32.0 ± 10.2**	**33.5 ± 10.8**	**35.1 ± 11.3**
LACI,%	16.7 ± 8.0	**22.8 ± 11.8**	**25.1 ± 13.9**	**21.0 ± 11.6**	**20.1 ± 10.3**

The comparisons with the no event population that were statistically significant with *p* < 0.05 are shown in bold type. *Estimated glomerular filtration rate (eGFR) was calculated by chronic kidney disease epidemiology collaboration (CKD-EPI) method. AA, African American; AF, atrial fibrillation; Ca, Caucasian; CHD, coronary heart disease; Ch, Chinese American; CVD, cardiovascular disease; HDL, high-density lipoprotein; HF, heart failure; Hi, Hispanic; LA, left atrium; LACI, left atrioventricular coupling index; EDVi, end-diastolic volume indexed; ESVi, end-systolic volume indexed; LV, left ventricle; LVEF, left ventricle ejection fraction; LVGFI, left ventricle global function index; LVMVR, LV mass/LV volume; NT-proBNP, N-terminal prohormone of brain natriuretic peptide.

Model discrimination was compared using C-statistic. The additional predictive value of the LACI was calculated using C-statistic increment, continuous net reclassification improvement (NRI), and integrative discrimination index (IDI) (25), and compared to the CHARGE-AF score (26), Framingham score (27), Agatston score (28), and LA or LV parameters. NRI and IDI were computed at 10 years using the R package “survIDINRI” (29). The survival tree method was used to determine the cut-off to transform the LACI into a binary variable with the best predictive value. Stratified analyses were performed in pre-specified subgroups defined by menopausal status and HT use at baseline (because estradiol levels differ substantially among women using versus not using HT) (6, 7). A two-tailed *p*-value < 0.05 was considered statistically significant. All data were analyzed using *R* software, version 3.6.1 (R Project for Statistical Computing).

## Results

### Study population and cardiovascular events

Among the 3,601 women in the MESA cohort, 2,087 (58.0%) underwent a baseline CMR examination and had available outcome data (mean age 61.2 ± 10.1 years). Of these women, 43.5% had hypertension, 11.4% were current smokers, 10.7% had diabetes mellitus, and their mean body mass index was 28.1 ± 5.6 kg/m^2^. At baseline, 35.9% of the women were on antihypertensive therapy, and 16.3% were on lipid-lowering medication. Among the 2,087 women, 415 (19.9%) were pre-menopausal and 1,672 (80.1%) were post-menopausal, including 1,110 (66.4%) using HT and 562 (33.6%) not using HT.

The population characteristics stratified by menopausal status and HT use are presented in Supplementary material 5. The baseline characteristics of the population, divided into those who developed AF (*n* = 176, 8.4%), hard CVD (*n* = 155, 7.4%), HF (*n* = 83, 4.0%), and CHD death (*n* = 71, 3.4%) over a mean follow-up period of 13.2 ± 3.3 years, are presented in Table 1. The mean follow-up was 11.3 ± 3.8 years for AF and 16.4 ± 1.1 years for other outcomes. When all clinical events were combined, 315 (15.1%) women had a CV event.

### Left atrioventricular coupling index distribution and relationship to menopausal status, hormone therapy use, and sex hormone levels

**FIGURE 2:**
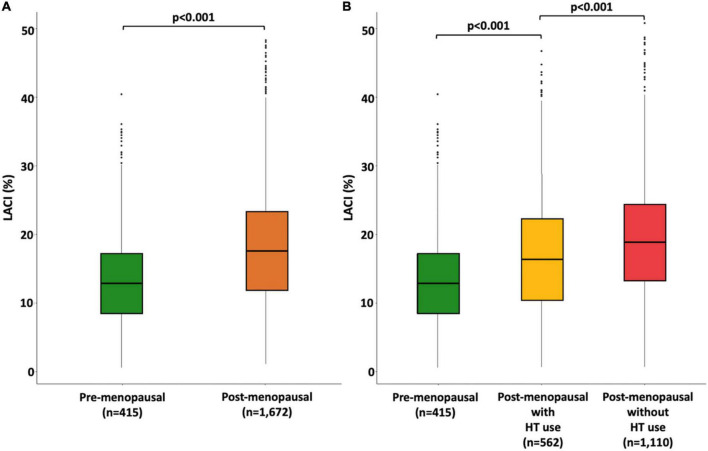
Boxplot distribution of the LACI measured at baseline stratified by menopause status **(A)** and by both menopausal status and hormone therapy use **(B)**. The bottom and top of the box are the first and third quartiles, and the band inside the box is the second quartile (the median).

**FIGURE 3:**
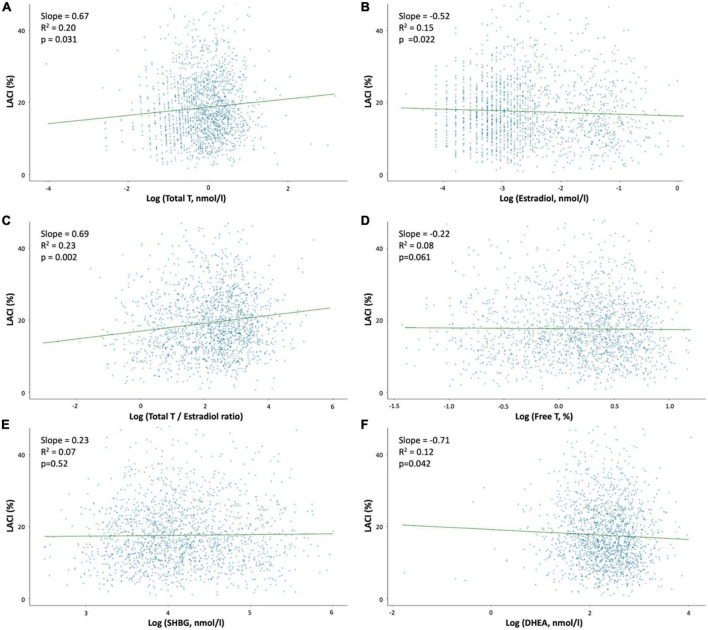
Association between the LACI value at baseline and sex hormone levels. Regression plot between LACI and log sex hormone levels in the overall population after adjustments for age, ethnicity, systolic blood pressure and diabetes mellitus. **(A)** Log (Total T, nmol/l); **(B)** Log (Estradiol, nmol/l); **(C)** Log (Testosterone/Estradiol ratio); **(D)** Log (Free T, nmol/l); **(E)** Log (SHBG, nmol/l); and **(F)** Log (DHEA, nmol/l).

In the entire population, the mean LACI was 17.6 ± 8.0%, and the first to third terciles of the LACI were ≤ 12.6, 12.6–19.5, and >19.5%, respectively (Supplementary material 6). The mean LACI was 16.7 ± 8.0% in women with no events. Pre-menopausal women had a lower LACI than post-menopausal women (15.0 ± 7.0 vs. 18.2 ± 8.7%, *p* < 0.001). Among post-menopausal women, women using HT had a lower LACI than women not using HT (17.0 ± 9.0 vs. 18.8 ± 8.0%, *p* < 0.001; Figure 2). Regarding sex hormone levels, a higher LACI value was associated with a higher total T level (*p* = 0.031), a higher total testosterone/estradiol ratio (*p* = 0.002), and lower estradiol (*p* = 0.022) and DHEA (*p* = 0.042) levels. The relationship between the LACI and free T, or SHBG levels at baseline were not statistically significant (Figure 3).

### Prognostic value of left atrioventricular coupling index in all women

**FIGURE 4:**
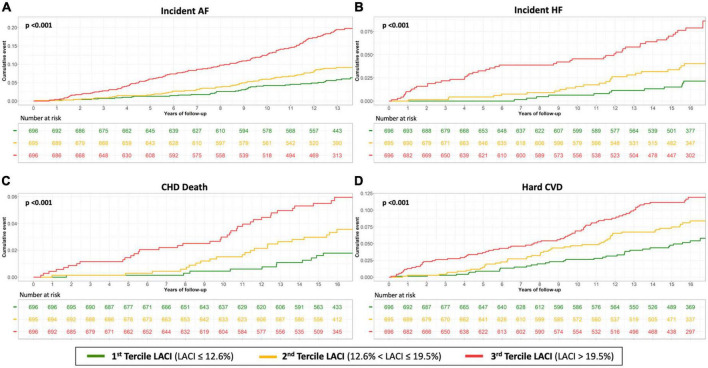
Kaplan–Meier survival curves for incident AF **(A)**, incident HF **(B)**, CHD death **(C)** and hard CVD **(D)** by LACI terciles in all women. The cumulative hazard was systematically significantly greater in the third quartile compared with the other terciles for each outcome (log-rank for difference; *p* < 0.001).

Unadjusted and adjusted Cox proportional hazard models for the LACI and the main LA and LV parameters are presented in Table 2 (other LA-LV parameters and biomarkers are presented in Supplementary material 7). After adjusting for risk factors, the LACI was positively associated with incident AF (hazard ratio [HR] 1.70; 95% CI 1.51–1.90), incident HF (HR 1.62; 95% CI 1.33–1.97), CHD death (HR 1.36; 95% CI 1.10–1.68), and hard CVD (HR 1.30; 95% CI 1.13–1.51; for all per one SD increment, *p* < 0.001). When the LACI was categorized in terciles, a LACI value > 19.5% was associated with incident AF, HF, CHD death, and hard CVD in comparison to the first tercile (≤12.6%, all *p* < 0.001; Figure 4). Using the survival tree method, the best LACI cut-offs for prediction were as follows: >28% to predict AF, >26% for HF, >25% for CHD death, and >26% for hard CVD. Using the composite outcome cut-off, including HF, AF, CHD death, and hard CVD, an increased LACI of >25% was independently associated with an increased occurrence of AF (HR 2.93; 95% CI 2.11–4.08, *p* < 0.001), HF (HR 2.45; 95% CI 1.54–3.91, *p* < 0.001), CHD death (HR 1.98; 95% CI 1.11–3.52, *p* = 0.023), and hard CVD (HR 1.59; 95% CI 1.11–2.29, *p* = 0.012; Table 2 and Supplementary material 8).

**TABLE 2:** Univariable and multivariable analysis of CV events occurrence according to left atrioventricular coupling index and other LA or LV parameters in all women (*n* = 2,087).

CMR parameters	Unadjusted			Adjusted*		
	HR (95%CI)	*p*-value	C-index (95%CI)	HR (95%CI)	*p*-value	C-index (95%CI)
**Incident AF**						
-LACI^†^,% (per 1 SD)	1.99 (1.73–2.13)	** < 0.001**	0.68 (0.64–0.72)	1.70 (1.51–1.90)	< 0.001	0.82 (0.80–0.85)
-LACI cut-off > 25%^‡^	4.18 (3.05–5.73)	** < 0.001**	0.62 (0.59–0.66)	2.93 (2.11–4.08)	** < 0.001**	0.80 (0.77–0.82)
-LA EDVi, ml/m^2^ (per 1 SD)	1.61 (1.46–1.79)	** < 0.001**	0.64 (0.60–0.68)	1.45 (1.31–1.61)	** < 0.001**	0.81 (0.78–0.83)
-LV EDVi, ml/m^2^ (per 1 SD)	0.86 (0.76–0.98)	**0.031**	0.57 (0.53–0.61)	0.97 (0.84–1.12)	0.67	0.79 (0.76–0.81)
-LVEF,% (per 1 SD)	0.98 (0.86–1.13)	0.82	0.50 (0.45–0.54)	0.98 (0.86–1.11)	0.73	0.79 (0.77–0.82)
-LV mass, g/m^2^ (per 1 SD)	1.03 (0.90–1.19)	0.63	0.50 (0.46–0.54)	0.99 (0.87–1.14)	0.92	0.79 (0.76–0.81)
-Peak LA strain,% (per 1 SD)	0.58 (0.49–0.64)	** < 0.001**	0.64 (0.60–0.68)	0.71 (0.60–0.83)	** < 0.001**	0.80 (0.77–0.82)
**Incident HF**						
-LACI^†^,% (per 1 SD)	2.05 (1.68–2.37)	** < 0.001**	0.71 (0.65–0.76)	1.62 (1.33–1.97)	** < 0.001**	0.84 (0.80–0.88)
-LACI cut-off > 25%^‡^	3.94 (2.54–6.10)	** < 0.001**	0.63 (0.58–0.69)	2.45 (1.54–3.91)	** < 0.001**	0.83 (0.79–0.87)
-LA EDVi, ml/m^2^ (per 1 SD)	1.77 (1.54–2.04)	** < 0.001**	0.69 (0.63–0.75)	1.56 (1.32–1.83)	** < 0.001**	0.83 (0.79–0.87)
-LV EDVi, ml/m^2^ (per 1 SD)	1.11 (0.89–1.38)	0.37	0.50 (0.43–0.58)	1.24 (0.99–1.56)	0.07	0.81 (0.77–0.85)
-LVEF,% (per 1 SD)	0.70 (0.59–0.81)	** < 0.001**	0.59 (0.52–0.67)	0.69 (0.59–0.81)	** < 0.001**	0.83 (0.79–0.87)
-LV mass, g/m^2^ (per 1 SD)	1.68 (1.40–2.00)	** < 0.001**	0.63 (0.56–0.69)	1.44 (1.18–1.75)	** < 0.001**	0.83 (0.79–0.87)
-Peak LA strain,% (per 1 SD)	0.61 (0.46–0.80)	** < 0.001**	0.63 (0.57–0.69)	0.74 (0.58–0.96)	**0.021**	0.82 (0.80–0.85)
**CHD death**						
-LACI^†^,% (per 1 SD)	1.80 (1.49–2.18)	** < 0.001**	0.67 (0.61–0.74)	1.36 (1.10–1.68)	** < 0.001**	0.87 (0.84–0.90)
-LACI cut-off > 25%^‡^	2.66 (1.62–4.38)	** < 0.001**	0.59 (0.54–0.65)	1.98 (1.11–3.52)	**0.023**	0.86 (0.83–0.90)
-LA EDVi, ml/m^2^ (per 1 SD)	1.48 (1.24–1.76)	** < 0.001**	0.62 (0.55–0.69)	1.28 (1.05–1.56)	**0.009**	0.87 (0.84–0.89)
-LV EDVi, ml/m^2^ (per 1 SD)	0.86 (0.69–1.07)	0.25	0.50 (0.43–0.58)	1.05 (0.82–1.33)	0.71	0.85 (0.82–0.88)
-LVEF,% (per 1 SD)	0.93 (0.74–1.17)	0.54	0.50 (0.42–0.57)	0.84 (0.67–1.05)	0.13	0.85 (0.82–0.89)
-LV mass, g/m^2^ (per 1 SD)	1.25 (1.00–1.55)	**0.049**	0.52 (0.44–0.60)	1.11 (0.89–1.40)	0.35	0.85 (0.82–0.89)
-Peak LA strain,% (per 1 SD)	0.83 (0.64–1.08)	0.17	0.57 (0.50–0.64)	0.99 (0.79–1.24)	0.93	0.85 (0.82–0.88)
**Hard CVD**						
-LACI^†^,% (per 1 SD)	1.65 (1.45–1.88)	** < 0.001**	0.64 (0.60–0.69)	1.30 (1.13–1.51)	** < 0.001**	0.78 (0.74–0.81)
-LACI cut-off > 25%^‡^	2.37 (1.68–3.34)	** < 0.001**	0.58 (0.54–0.61)	1.59 (1.11–2.29)	**0.012**	0.76 (0.73–0.80)
-LA EDVi, ml/m^2^ (per 1 SD)	1.33 (1.16–1.52)	** < 0.001**	0.60 (0.55–0.64)	1.19 (1.04–1.36)	**0.014**	0.77 (0.74–0.80)
-LV EDVi, ml/m^2^ (per 1 SD)	0.93 (0.81–1.06)	0.45	0.55 (0.51–0.60)	0.88 (0.74–1.05)	0.15	0.76 (0.72–0.79)
-LVEF,% (per 1 SD)	0.86 (0.74–1.00)	**0.045**	0.55 (0.51–0.60)	0.83 (0.72–0.96)	**0.011**	0.76 (0.72–0.80)
-LV mass, g/m^2^ (per 1 SD)	1.15 (0.98–1.34)	0.08	0.51 (0.46–0.56)	1.05 (0.89–1.23)	0.33	0.76 (0.72–0.79)
-Peak LA strain,% (per 1 SD)	0.88 (0.74–1.05)	0.15	0.55 (0.51–0.60)	1.00 (0.86–1.17)	0.98	0.76 (0.72–0.79)

*Adjusted model included age, ethnicity, education level, physical activity, menopausal status, hormone therapy (HT) use, diabetes mellitus, current smoking, systolic blood pressure, anti-hypertensive therapy, body mass index, high-density lipoprotein cholesterol, total cholesterol, lipid-lowering therapy, total testosterone/estradiol ratio, DHEA level, and glomerular filtration rate.

^†^LACI used as continuous variable.

^‡^LACI used as binary variable defined by a cut-off >25%. -All LV parameters, LA parameters and LACI values were normalized according to the following formula: (parameter–mean value)/standard. AF, atrial fibrillation; CHD, coronary heart disease; CI, confidence interval; CVD, cardiovascular disease; HF, heart failure; HR, hazard ratio; LA, left atrium; LACI, left atrioventricular coupling index; EDVi, end-diastolic volume indexed; ESVi, end-systolic volume indexed; LV, left ventricle; LVEF, left ventricle ejection fraction.

Bold represent the statistically significant results.

### Prognostic value of left atrioventricular coupling index according to menopausal status and hormone therapy use

When the best LACI cut-off was used, it was found that post-menopausal women with a LACI > 25% presented a higher risk of AF, HF, CHD death, and hard CVD than pre-menopausal women or post-menopausal women with a LACI ≤ 25% (*p* < 0.001, Supplementary material 9). As already mentioned, to account for the fact that estradiol levels differ considerably between women using HT and those not using HT, we performed stratified analyses to compare pre-menopausal women and post-menopausal women using HT to post-menopausal women not using HT. The global prognostic value of the LACI was homogeneous in these two groups (Table 3).

**TABLE 3:** Univariable and multivariable analysis of left atrioventricular coupling index for prediction of CV events stratified by menopausal status (*n* = 2,087).

Outcomes	Unadjusted			Adjusted		
	HR (95%CI)	*p*-value	C-index (95%CI)	HR (95%CI)	*p*-value	C-index (95%CI)
**Incident AF**						
-LACI* in pre-menopausal women or post-menopausal women with HT use (*n* = 977)	1.98 (1.42–2.52)	** < 0.001**	0.68 (0.62–0.74)	1.68 (1.18–1.58)	**0.003**	0.82 (0.80–0.85)
-LACI* in post-menopausal women without HT use (*n* = 1,110)	2.02 (1.56–2.36)	** < 0.001**	0.69 (0.64–0.73)	1.73 (1.34–1.73)	** < 0.001**	0.83 (0.81–0.85)
**Incident HF**						
-LACI* in pre-menopausal women or post-menopausal women with HT use (*n* = 977)	1.82 (1.12–2.07)	**0.004**	0.70 (0.62–0.77)	1.38 (1.03–1.79)	**0.022**	0.83 (0.77–0.88)
-LACI* in post-menopausal women without HT use (*n* = 1,110)	2.22 (1.63–2.33)	** < 0.001**	0.72 (0.64–0.78)	1.77 (1.23–2.28)	** < 0.001**	0.85 (0.78–0.89)
**CHD death**						
-LACI* in pre-menopausal women or post-menopausal women with HT use (*n* = 977)	1.82 (1.21–2.50)	** < 0.001**	0.67 (0.59–0.76)	1.32 (1.02–1.76)	**0.034**	0.86 (0.80–0.92)
-LACI* in post-menopausal women without HT use (*n* = 1,110)	1.77 (1.26–2.35)	** < 0.001**	0.67 (0.60–0.76)	1.30 (1.04–1.70)	**0.013**	0.86 (0.81–0.91)
**Hard CVD**						
-LACI* in pre-menopausal women or post-menopausal women with HT use (*n* = 977)	1.50 (1.15–1.88)	**0.003**	0.63 (0.58–0.69)	1.28 (1.03–1.81)	**0.044**	0.76 (0.71–0.80)
-LACI* in post-menopausal women without HT use (*n* = 1,110)	1.72 (1.42–2.39)	** < 0.001**	0.65 (0.60–0.70)	1.38 (1.09–1.98)	**0.025**	0.78 (0.72–0.81)

*LACI used as continuous variable, % (per 1 SD). -LACI values were normalized according to the following formula: (parameter–mean value)/standard deviation. Adjusted model included age, ethnicity, education level, physical activity, diabetes mellitus, current smoking, systolic blood pressure, anti-hypertensive therapy, body mass index, high-density lipoprotein cholesterol, total cholesterol, lipid-lowering therapy, total testosterone/estradiol ratio, DHEA level, and glomerular filtration rate. AF, atrial fibrillation; CHD, coronary heart disease; CI, confidence interval; CVD, cardiovascular disease; HF, heart failure; HR, hazard ratio; LACI, left atrioventricular coupling index.

Bold represent the statistically significant results.

**FIGURE 5:**
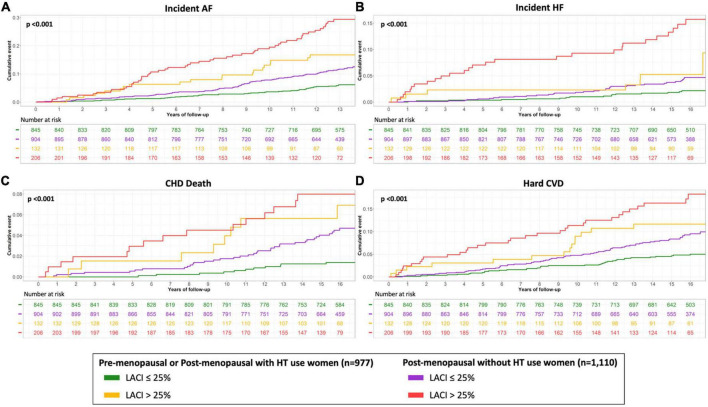
Kaplan–Meier survival curves for incident AF **(A)**, incident HF **(B)**, CHD death **(C)** and hard CVD **(D)** according to the menopausal status and HT use stratified by LACI > 25%. The cumulative hazard was systematically significantly greater in the third quartile compared with the other terciles for each outcome (log-rank for difference; *p* < 0.001).

In women with a LACI > 25%, the cumulative hazard was higher for post-menopausal women not using HT than for pre-menopausal women or post-menopausal women using HT (*p* < 0.001). Regardless of whether the women were pre-menopausal, menopausal and not using HT, or post-menopausal and not using HT, the cumulative hazard was higher for women with a LACI > 25% than for women with a LACI ≤ 25% (*p* < 0.001; Figure 5).

### Improvement in risk prediction with the addition of left atrioventricular coupling index

The multivariable model with the LACI showed significant improvement in model discrimination and reclassification compared to the model with traditional risk factors for predicting AF (C-statistic: 0.82 vs. 0.79; NRI = 0.325; IDI = 0.036), HF (C-statistic: 0.84 vs. 0.81; NRI = 0.571; IDI = 0.023), CHD death (C-statistic: 0.87 vs. 0.85; NRI = 0.506; IDI = 0.012), and hard CVD (C-statistic: 0.78 vs. 0.76; NRI = 0.229; IDI = 0.012). The LACI also had better discrimination and reclassification for these outcomes than the model with each LA or LV parameter and the other biomarkers and scores (Table 2 and Supplementary material 10).

## Discussion

This study focused on a large multi-ethnic population of pre- and post-menopausal women aged 45–84 years who were free of clinical CVD at baseline. The participants were followed for more than 13 years. The main findings were as follows: (i) the LACI showed the greatest association with incident AF, HF, CHD death, and hard CVD, improving model discrimination and reclassification of cardiovascular event risk above menopausal status, traditional cardiovascular risk factors, and sex hormone levels; (ii) the prognostic value of the LACI was homogeneous in both pre-menopausal and post-menopausal women; (iii) post-menopausal women had worse left atrioventricular function, defined by a higher LACI value, than pre-menopausal women; (iv) post-menopausal women using HT had better left atrioventricular function, defined by a lower LACI value, than post-menopausal women not using HT; and (v) the LACI was positively associated with total testosterone and DHEA levels, but inversely associated with estradiol level. To our knowledge, the prognostic value of a left atrioventricular coupling index in pre- and post-menopausal women and the associaton of LACI and sex hormone levels have not been previously well-established.

In CVD pathophysiology, specific biological mechanisms have been described in women due to the role of endogenous sex hormones (1, 5, 6, 8, 30). However, our analysis of pre- and post-menopausal women has shown that the LACI has a higher prognostic value and improves model discrimination and reclassification in predicting the risk of outcomes compared to individual LA or LV parameters. These features are maintained even after adjusting for traditional risk factors, menopausal status, HT use, and sex hormone levels (total testosterone/estradiol ratio). Therefore, this study highlights the prognostic importance of atrioventricular coupling, reflected by the intricate hemodynamic interactions between the left atrium and ventricle during LV diastole (31), in stratifying cardiovascular event risk in peri- and post-menopausal women.

We also investigated the best LACI cut-off threshold, and found that a LACI > 25% was the best cut-off for the composite of all four cardiovascular outcomes. This is in line with the prior study that defined the LACI in all the MESA population, including both men and women (15). Moreover, the normal value of LACI already published is 16 ± 8% (15). Interestingly, regardless of whether women were menopausal and not using HT, or pre-menopausal or post-menopausal not using HT, the risk of outcomes was higher for women with a LACI > 25% than for women with a LACI ≤ 25%. In contrast, among all women with a LACI > 25%, post-menopausal women not using HT had a higher outcomes rate than pre-menopausal women or post-menopausal women using HT. All these findings support the notion that the LACI has an additional prognostic value above that of menopausal status and HT use; however, menopausal status and HT use also have important prognostic value beyond the LACI.

In this study, when the LACI value was used as a marker of left atrioventricular function (19), it was found that post-menopausal women had worse left atrioventricular function than pre-menopausal women (18.2 vs. 15.0%). Given that menopause is also a marker of aging, these findings are consistent with those of a prior study that used CMR imaging of 40 healthy individuals, including 17 women, to investigate the effects of aging on left atrioventricular coupling (14). The oldest participants had larger LA volumes, smaller LV volumes, and larger LA/LV end-diastolic volume ratios (27 ± 6 vs. 19 ± 3%; *p* < 0.001).

Beyond menopausal status, we also assessed left atrioventricular function according to HT use, because estradiol levels differ substantially among women who use HT and those who do not. This analysis showed that among post-menopausal women, those using HT had better left atrioventricular function, defined by a lower LACI value, than women not using HT. However, the significant difference in LACI values between pre-menopausal women and post-menopausal women using HT suggests that HT may not exert the same physiological effects as endogenous sex hormones. Consistently, some studies have reported the effects on CVD risk of formulations of estrogen and progestin and the time since HT initiation (32). Beyond the previously described effects of sex hormones on LV structure (7), these findings suggest a potential additional effect of sex hormones on left atrioventricular coupling in peri- and post-menopausal women.

Regarding the association between sex hormone levels and heart structure, recent studies have shown that higher free testosterone and lower SHBG levels are associated with greater LV mass and an increased LV mass-volume-ratio (concentric remodeling) (7), higher endothelial dysfunction assessed using brachial artery flow-mediated dilation (33), higher LV diastolic dysfunction (34), and greater coronary artery calcium score progression (35). However, total testosterone and estradiol levels have not been associated with LV structure (7). Despite the contradictory outcomes of prospective studies that have assessed the plausible pathological pathways that link endogenous sex hormones and clinical CVD events, the MESA has recently shown that in post-menopausal women, a higher total testosterone level and a lower estradiol level are associated with an increased risk of CVD (6). However, the study also found that free testosterone and SHBG levels are not prognosticators of CVD (6). Together, these results suggest that total testosterone and estradiol levels influence CVD risk via mechanisms other than modification of LV structure.

In the current study, a higher LACI value was significantly associated with a higher DHEA level, a higher total testosterone level, a lower estradiol level, and thus a higher total testosterone/estradiol ratio. Based on these results, we hypothesize that these sex hormones influence the left atrioventricular coupling function. Indeed, estradiol can downregulate angiotensin receptors, improve vascular endothelium function, protect against vascular injury, and inhibit adverse LV remodeling processes (8). In contrast, testosterone can increase platelet aggregation via upregulation of thromboxane and induce vasoconstriction (36). The effect of DHEA on CVD has not yet been defined. It was hypothesized that lower DHEA levels may be associated with an increased risk of cardiovascular events (37); however, this association has been disproved by numerous studies, particularly those conducted with elderly women (38). Therefore, in addition to these reported mechanisms by which estradiol and testosterone may play a prognostic role for peri- and post-menopausal women, the current findings suggest that the abrupt loss of estradiol at menopause and the potential increase in total testosterone may be significant contributors to left atrioventricular coupling. Moreover, the LACI was not associated with free testosterone and SHBG levels in this study, which supports the hypothesis that total testosterone and estradiol influence CVD risk through effects on left atrioventricular coupling and not just the modification of LV structure.

Because the LACI is an independent prognosticator of cardiovascular events, even after adjusting for the total testosterone/estradiol ratio, the association between the LACI and both the total testosterone and estradiol levels supports the hypothesis that these hormones impact left atrioventricular coupling. Therefore, all these findings emphasize the complexity of the female hormonal system during the peri- and post-menopausal states; there are hormones acting independently and in combination. The findings suggest that the abrupt modification of sex hormone levels during menopause could critically influence left atrioventricular coupling, thus explaining the increase in cardiovascular events in older women. Beyond menopausal status and sex hormone levels, the LACI may help identify women who have an increased CVD risk and who may benefit from additional risk-reducing strategies.

### Study limitations

This study has several limitations. First, the general applicability of these findings may be limited by survivor biases. The women who participated had no known CVD at baseline; therefore, the older women undergoing CMR in this cohort represent a healthier sample than the general older population. Second, the LACI was investigated as a primary prevention tool in the early detection of CVD risk in asymptomatic women without CVD. Therefore, the LACI would not be the ideal parameter for assessing a woman with CVD and/or both LA and LV enlargements. Third, menopausal status was self-reported, which introduces a risk of reporting bias. To minimize this bias we corrected self-reported menopausal status using an algorithm based on age, self-reported hysterectomy, bilateral oophorectomy, and menopausal age (6). Moreover, a single measurement of sex hormones was performed at baseline, by radioimmunoassay and not mass spectrometry, which is the current gold standard. Fourth, two-dimensional instead of three-dimensional methods were used to measure LA volumes, which may have underestimated volumes by 11.5–20% (39). Nevertheless, this method has been validated (22, 23). In addition, echocardiographical data were not collected in MESA study. Fifth, this study was designed to assess the prognostic impact of different parameters on the occurrence of incident AF, but the management of AF by ablation or medical treatment of these patients was not collected. Sixth, the optimal cut-off value for LACI was not validate using an external validation cohort. However, to our knowledge, there is no existing cohort of peri-menopausal women without known CVD with CMR at baseline and with such an important median long-term follow-up. Further studies will therefore need to validate these results. Since CMR is not widely accessible, the use of the LACI as a screening tool in the general population should be investigated using echocardiography.

## Conclusion

In a large multi-ethnic study population of pre-menopausal and post-menopausal women who were free of clinical CVD at baseline, a higher LACI (measured using CMR imaging) was associated with a higher risk of incident AF, HF, CHD death, and hard CVD during a 13-year average follow-up and this association did not vary by menopause status. Beyond menopausal status and HT use, the addition of the LACI to risk prediction models for these outcomes showed improvements in model discrimination and reclassification of cardiovascular event risks. Finally, the association between the LACI and total testosterone and estradiol levels suggests a potential effect of these hormones on left atrioventricular coupling, which should be confirmed infurther studies.

## Data availability statement

The raw data supporting the conclusions of this article will be made available by the authors, without undue reservation.

## Ethics statement

The studies involving human participants were reviewed and approved by IRB of MESA study. The patients/participants provided their written informed consent to participate in this study.

## Author contributions

TP and JL conceived the study design. TP, EM, BV, and JL analyzed data and drafted the manuscript with critical revision. As authors, we attest to each of our substantial contributions to the manuscript and revision. All authors participated in the discussion of the concept of the study and read and approved the final manuscript.

## Footnotes

1
www.mesa-nhlbi.org

## Abbreviations

AF: atrial fibrillation
CHD: coronary heart disease
CMR: cardiovascular magnetic resonance
CVD: cardiovascular disease
DHEA: dehydroepiandrosterone
HF: heart failure
HT: hormone therapy
IDI: integrative discrimination index
LA: left atrial
LACI: left atrioventricular coupling index
LV: left ventricular
LVEF: left ventricular ejection fraction
MESA: Multi-Ethnic Study of Atherosclerosis
NRI: net reclassification improvement
SHBG: sex hormone binding globulin.
