# Supplementary material for: Prognostic value of a left atrioventricular coupling index in pre- and post-menopausal women from the Multi-Ethnic Study of Atherosclerosis
Source: Front Cardiovasc Med. 2022 Nov 21;9:1066849. doi: 10.3389/fcvm.2022.1066849 (PMC9719991; doi:10.3389/fcvm.2022.1066849)
Supplement: Supplementary file 1 [file Data_Sheet_1.docx]

**SUPPLEMENTS**

**Table of Contents:**

- Supplement 1: Methodology of baseline characteristics collection.
- Supplement 2: Cardiac MRI protocol and analysis.
- Supplement 3: Methodology of LA measurements by CMR.
- Supplement 4: Definition of outcomes.
- Supplement 5: Population characteristics of women at baseline according to the menopausal status and hormone therapy use.
- Supplement 6: Distribution of LACI.
- Supplement 7: Univariable and multivariable analysis of CV events occurrence according to LACI and other LA/LV parameters or biomarkers and scores.
- Supplement 8: Kaplan-Meier survival curves for incident AF (A), incident HF (B), CHD death (C) and hard CVD (D) stratified by LACI >25%.
- Supplement 9: Kaplan-Meier survival curves for incident AF (A), incident HF (B), CHD death (C) and hard CVD (D) according to the menopausal status and stratified by LACI >25%.
- Supplement 10: Discrimination and reclassification associated with LACI to different LA, LV parameters, biomarker and scores.

**SUPPLEMENT 1:**

**Methodology of baseline characteristics collection**

- **Standardized questionnaires** were used at baseline (Exam 1) to collect information about age, sex, race/ethnic background and cigarette smoking. A standardized medication inventory including hormone therapy was used to collect information on prescription and nonprescription medications.
- **Level of education** was classified as <high school, high school/technical school/associate degree, and college/graduate/professional school.
- **Physical activity** was estimated as the total amount of intentional moderate or vigorous exercise performed in a usual week, and measured in metabolic equivalent task-minutes.
- **Cigarette smoking** was categorized as current, former or never.
- **Body mass index** was calculated as weight divided by height squared (kg/m^2^) with weight measured to the nearest 0.5kg and height to the nearest 0.1 cm.
- **Blood pressure** was measured 3 times using a Dinamap model Pro 100 automated oscillometric sphygmomanometer (Critikon, Tampa, FL) while the participants were resting in a seated position. The average of the last two measurements were used in the analysis. Fasting glucose was obtained by a thin-film adaptation of the glucose oxidase method (Johnson & Johnson Clinical Diagnostics, Inc, Rochester, NY).
- **Blood samples** were obtained from participants at the baseline examination after a 12-h fast. Details of measurements of clinical chemistry parameters are already described.^1^ Diabetes mellitus was defined as a fasting glucose of ≥126 mg/dL or use of hypoglycemic medication. Low-density lipoprotein cholesterol was calculated with the Friedewald equation. Serum creatinine was measured using colorimetry with a Johnson & Johnson Vitros 950 analyser (Johnson & Johnson Clinical Diagnostics, Rochester, New York, USA) and calibrated to the Cleveland Clinic standard. Estimated glomerular filtration rate (eGFR) was calculated on the basis of serum creatinine and cystatin C concentrations using the CKD-EPI equation.^2^
- **Baseline sex hormone concentrations** were measured from fasting serum samples that were drawn at the baseline examination between 7:30 AM and 10:30 AM and stored at −70 °C until analysis. Hormone assays were performed at the University of Massachusetts Medical Center in Worcester, MA. E2 was measured using an ultrasensitive radio-immunoassay kit (Diagnostic System Laboratories, Webster, TX). Total T and dehydroepiandrosterone (DHEA) were measured using radio-immunoassay kits, and SHBG was measured by chemiluminescence enzyme immunometric assay using Immulite kits (Diagnostic Products Corporation, Los Angeles, CA).^3^ Free T was calculated using the Sodergard method,^4^ and reported as percent of total T. We divided total testosterone by estradiol to calculate total testosterone/estradiol ratio for each participant. Quality control serum was obtained from an about 10% blind pool. The intra-assay coefficients of variation for total T, SHBG, DHEA, and E2 were 12.3%, 9.0%, 11.2%, and 10.5%, respectively.^5^

References for Supplemental Methods:

1. Ouyang P, Vaidya D, Dobs A, Golden SH, Szklo M, Heckbert SR, et al. Sex hormone levels and subclinical atherosclerosis in postmenopausal women: the Multi-Ethnic Study of Atherosclerosis. Atherosclerosis. 2009;204(1):255–61.
2. Inker LA, Schmid CH, Tighiouart H, Eckfeldt JH, Feldman HI, Greene T, et al. Estimating glomerular filtration rate from serum creatinine and cystatin C. N Engl J Med. 2012;367(1):20–9.
3. Golden SH, Dobs AS, Vaidya D, Szklo M, Gapstur S, Kopp P, et al. Endogenous sex hormones and glucose tolerance status in postmenopausal women. J Clin Endocrinol Metab. 2007;92(4):1289–95.
4. Södergård R, Bäckström T, Shanbhag V, Carstensen H. Calculation of free and bound fractions of testosterone and estradiol-17 beta to human plasma proteins at body temperature. J Steroid Biochem. 1982;16(6):801–10.
5. Laughlin GA, Goodell V, Barrett-Connor E. Extremes of endogenous testosterone are associated with increased risk of incident coronary events in older women. J Clin Endocrinol Metab. 2010;95(2):740–7.

**SUPPLEMENT 2:**

**Cardiac MRI protocol and analysis.**

1. ***Cardiac MRI Protocol***

Cardiac MR examinations consisted of short- and long-axis cine images, phase contrast images of the aorta, and black blood aorta images. The protocol was accomplished within 30–45 min. All images were acquired during short breath-holding (12–15 sec) at resting lung volume.

First, four midline ECG-gated sagittal scout images were acquired with a fast gradient-echo sequence (series 1). These images were used to confirm correct positioning of the phased-array surface coil. Second, the same sequence was used to acquire three axial scout views (series 2); these images were acquired beginning 2 cm above the diaphragm. Next, a pseudovertical long-axis scout image (series 3) was obtained using the largest LV image from series 2. This slice extended through the middle of the mitral valve plane and the LV apex. Four-chamber long-axis cine images (series 4) were next acquired using a cine ECG-gated fast gradient-echo pulse sequence. The imaging plane for the four-chamber view was prescribed along points intersecting the middle of the mitral valve plane and the LV apex.

Short-axis cine images (series 5) were acquired from the end-diastolic image of the four-chamber acquisition, by prescribing 10–12 slices perpendicular to a line from the middle of the mitral valve plane to the cardiac apex. A two-chamber vertical long-axis cine sequence (series 6) was obtained by prescribing a single slice along a line extending from the LV apex to the middle of the mitral valve plane as viewed on the four-chamber view (series 4). The image prescription was designed to minimize variation among the MR field centers. Cine images were obtained with a temporal resolution of approximately 50 msec or less.

1. ***Cardiac MRI Analysis***

Cardiac MRI were performed at 6 MESA field centers using 1.5‐Tesla scanners, either Signa LX or CVi (GE Medical Systems) or Symphony or Sonata (Siemens Medical Systems). Cardiac MR images were transmitted using DICOM transfer protocol to the central cardiac MR review center in Baltimore, MD, at Johns Hopkins Hospital. The cardiac MR images were transferred to a workstation (UNIX, Sun Microsystems) for analysis. Image data were analyzed using MASS software ([version 4.2] Medis). Images were magnified to 250%. Image contrast was set to 60; image brightness was set to 60; window width and level were set using the Auto function in MASS, which sets the maximum and minimum pixel values in the displayed image to values of 255 and 0, respectively.

Image analysts were trained technologists who received lectures in cardiac anatomy and function. Technologists were trained on a set of 40 training cases that were reviewed by an experienced cardiac MR physician. The training period examinations were accepted if LV function parameters were within 10% of the physician-determined values. The endocardial and epicardial borders were traced semiautomatically at both end-diastole and end-systole on short-axis cine images and were then corrected manually at the base of the heart. Corrections to other images were allowed if visual inspection revealed obviously incorrect borders. The papillary muscles were included in the LV end-diastolic volume and LV end-systolic volume and excluded from the LV mass. All image contours were checked by a cardiac MR physician after contouring was finished by the technologist.

LV end-diastolic volume and LV end-systolic volume were calculated using Simpson's rule (the summation of areas on each separate slice multiplied by the sum of slice thickness and image gap). LV mass was determined by the sum of the myocardial area (the difference between endocardial and epicardial contour) times slice thickness plus image gap in the end-diastolic phase multiplied by the specific gravity of myocardium (1.05 g/mL). LV stroke volume was calculated as the difference between LV end-diastolic volume and LV end-systolic volume. LVEF was calculated as LV stroke volume divided by LV end-diastolic volume multiplied by 100. Indexed parameters (e.g., LV mass index) were calculated by dividing each parameter (e.g., LV mass) by body surface area.

The central cardiac MR review center in Baltimore, MD, at Johns Hopkins Hospital, blinded to the participant’s case status, defined endocardial and epicardial borders of the LA at end-systole. Using the marked points, the software creates endocardial and epicardial borders, then tracks LA tissue in subsequent frames. The endocardial and epicardial contours generated by the software are then followed by the operator during the cardiac cycle for quality control.

**SUPPLEMENT 3:**

**Methodology of LA measurements by CMR**

Maximum, pre-atrial, and minimum contraction left atrium (LA) volumes were extracted from volume curves that were created using the area-length method from apical 2- and 4-chamber views, using the following formula for Biplane calculation:$Volume= \frac{(0.0848 \times area\text{4ch }\times area\text{4ch})}{(\left[ \mathrm{length}\text{4ch}+length\text{2ch} \right]/2)}$.

All LA volumes were indexed to body surface area (ml/m^2^). Biplanar volume and function assessment on MTT had a strong positive linear correlation and concordance to other manual methods (e.g. Simpson’s method).

The software calculates global longitudinal atrial strain by averaging longitudinal strain of all LA segments in 2- and 4-chamber views during each cardiac cycle. Global peak longitudinal LA strain (peak LA strain) was measured from the global longitudinal strain curve.

The LA cycle has been described in 3 phases, a reservoir collecting pulmonary venous flow during ventricular systole, a conduit for passage of blood into the left ventricle during early diastole and a booster pump by augmenting ventricular filling during late diastole via atrial contraction.

The LA volume indexed variables were:

- LA end-systolic volume (maximum LA volume): before mitral valve opening.
- LA end-diastolic volume (minimum LA volume): after mitral valve closure.

**SUPPLEMENT 4:**

**Definition of outcomes**

***Heart Failure (HF)***

The diagnosis of HF required ≥1 criterion such as pulmonary edema, congestion by chest X-ray; dilated ventricle or poor LV function by echocardiography or ventriculography; or evidence of left diastolic dysfunction, in addition to symptoms and HF diagnosed by a physician. Moreover, we included both probable and definite diagnosis of HF.

***Coronary Heart Disease (CHD) death***

Definite fatal CHD required a documented myocardial infarction (MI) within the previous 28 days, chest pain within the 72 hours before death, or a history of CHD, and required the absence of a known non-atherosclerotic or non-cardiac cause of death. If the definite fatal CHD criteria were not met, possible fatal CHD could be assigned with an underlying cause of death consistent with fatal CHD and required the absence of a known non-atherosclerotic or non-cardiac cause of death.

***Atrial Fibrillation (AF)***

Incident cases of AF during the follow-up period were identified through MESA surveillance and, for participants enrolled in fee-for-service Medicare, from inpatient and outpatient Medicare claims data. As a part of standard event surveillance procedures, all hospitalizations were identified during follow-up calls to study participants or a proxy. Discharge diagnosis and procedure codes from those hospitalizations were abstracted. AF was documented as present if an International Classification of Diseases, Ninth Revision diagnosis code 427.31 (AF) or 427.32 (atrial flutter) was present.

***Myocardial Infarction (MI)***

The diagnosis of MI required either abnormal cardiac biomarkers (two times upper limits of normal) regardless of pain or ECG findings; evolving Q waves regardless of pain or biomarker findings; or a combination of chest pain, and ST-T evolution or new left bundle branch block (LBBB), and biomarker levels 1-2 times upper limits of normal.

***Resuscitated Cardiac Arrest***

Reviewers classified resuscitated cardiac arrest when a patient successfully recovered from a full cardiac arrest through cardiopulmonary resuscitation (including cardioversion), or evidence of ischemia by stress tests or by resting ECG. We considered coronary revascularization or a physician diagnosis of angina or CHD, in the absence of symptoms, to not be angina.

***Stroke***

Stroke was classified as present or absent and consisted of rapid onset of a documented focal neurologic deficit lasting 24 hours or until death, or if < 24 hours, there was a clinically relevant lesion on brain imaging. Patients with focal neurologic deficits secondary to brain trauma, tumor, infection, or other non-vascular cause were excluded.

**SUPPLEMENT 5:**

**Table. Population characteristics of women at baseline according to the menopausal status and hormone therapy use (n=2,087).**

|  |  |  | **Post-menopausal women** | | |
| --- | --- | --- | --- | --- | --- |
| **Baseline parameters** | **All women (n=2,087)** | **Pre-menopausal women**  **(n=415)** | **All**  **(n=1,672)** | **With HT use**  **(n=562)** | **Without HT use**  **(n=1,110)** |
|  |  |  |  |  |  |
| Age, years | 61.2 ± 10.1 | 50.3 ± 6.3 | **63.9 ± 9.0** | **61.7 ± 8.4** | **65.0 ± 9.1** |
| Ethnicity (Ca/Ch/AA/Hi), % | 39/12/27/21 | 37/13/29/20 | **40/12/27/21** | **59/8/19/15** | **30/14/31/25** |
| Education |  |  |  |  |  |
| < High school | 363 (17.4) | 46 (11.1) | **317 (19.0)** | **62 (11.0)** | **255 (23.0)** |
| High school, technical school, or  associate degree | 1040 (49.8) | 195 (47.0) | **845 (50.5)** | **282 (50.2)** | **563 (50.7)** |
| College, graduate or professional  school | 684 (32.8) | 174 (41.9) | **510 (30.5)** | **218 (38.8)** | **292 (26.3)** |
| Hypertension, n (%) | 907 (43.5) | 88 (21.2) | **819 (49.0)** | **306 (54.4)** | **547 (49.3)** |
| Systolic blood pressure, mmHg | 126 ± 23 | 115 ± 18 | **129 ± 24** | **126 ± 23** | **130 ± 24** |
| Diastolic blood pressure, mmHg | 69 ± 10 | 69 ± 8 | 69 ± 10 | 68 ± 10 | **70 ± 11** |
| Body mass index, kg/m^2^ | 28.1 ± 5.6 | 28.2 ± 6.0 | 28.0 ± 5.5 | **27.4 ± 5.5** | 28.4 ± 5.5 |
| Diabetes mellitus, n (%) | 223 (10.7) | 30 (7.2) | **193 (11.5)** | 41 (7.3) | **152 (13.7)** |
| Current Smoking, n (%) | 237 (11.4) | 57 (13.7) | **180 (10.8)** | **64 (11.4)** | **116 (10.5)** |
| Heart rate, bpm | 64 ± 9 | 64 ± 9 | 64 ± 9 | 64 ± 9 | 64 ± 9 |
| Total cholesterol, mg/dl | 200 ± 34 | 190 ± 32 | **202 ± 34** | **199 ± 31** | **204 ± 36** |
| HDL cholesterol, mg/dl | 57 ± 15 | 55 ± 15 | **57 ± 15** | **62 ± 16** | 55 ± 15 |
| eGFR**^*^**, ml/min/1.73m^2^ | 78.4 ± 16.2 | 86.9 ± 15.0 | **76.2 ± 15.8** | **77.5 ± 15.4** | **75.6 ± 16.0** |
| Hypertension medication, n (%) | 750 (35.9) | 80 (19.3) | **670 (40.1)** | **229 (40.7)** | **441 (39.7)** |
| Lipid-lowering medication, n (%) | 340 (16.3) | 26 (6.3) | **314 (18.8)** | **99 (17.6)** | **215 (19.4)** |
| NT-proBNP, pg/ml | 107 (37-178) | 64 (24-111) | **118 (63-222)** | **122 (67-221)** | **114 (60-222)** |
| Agatston score, mean ± SD | 65 ± 199 | 12 ± 64 | **78 ± 218** | **55 ± 161** | **90 ± 241** |
| Framingham CVD risk, % | 10.0 ± 8.0 | 4.8 ± 4.6 | **11.2 ± 8.2** | **9.4 ± 7.3** | **12.2 ± 8.4** |
| CHARGE-AF score | 11.7 ± 1.2 | 10.4 ± 0.8 | **12.0 ± 1.1** | **11.8 ± 1.0** | **12.0 ± 1.1** |
| Baseline LV characteristics | | | | | |
| LV EDVi, ml/m^2^ | 65.4 ± 12.4 | 68.4 ± 11.4 | **64.7 ± 12.6** | **65.3 ± 12.5** | **64.4 ± 12.6** |
| LVEF, % | 63.6 ± 5.6 | 63.5 ± 5.2 | 63.7 ± 5.7 | 63.8 ± 5.6 | 63.6 ± 5.8 |
| LV mass index, g/m^2^ | 58.2 ± 10.5 | 57.4 ± 9.0 | **58.4 ± 10.8** | 57.6 ± 10.2 | **58.9 ± 11.1** |
| LVMVR, g/ml | 0.90 ± 0.15 | 0.85 ± 0.13 | **0.92 ± 0.16** | **0.89 ± 0.14** | **0.93 ± 0.16** |
| LVGFI, % | 41.7 ± 5.9 | 43.0 ± 5.7 | **41.4 ± 5.9** | **42.0 ± 5.8** | **41.1 ± 6.0** |
| Baseline LA characteristics | | | | | |
| LA EDVi, ml/m^2^ | 11.1 ± 5.9 | 10.0 ± 5.2 | **11.4 ± 6.1** | **11.1 ± 6.3** | **11.6 ± 6.0** |
| LA ESVi, ml/m^2^ | 29.1 ± 9.4 | 28.8 ± 8.2 | 29.3 ± 9.7 | 29.2 ± 10.0 | 29.3 ± 9.6 |
| Peak LA strain, % | 38.3 ± 10.9 | 40.3 ± 11.1 | **37.8 ± 10.7** | 39.3 ± 11.1 | **37.1 ± 10.5** |
| LACI, % | 17.6 ± 8.0 | 15.0 ± 7.0 | **18.2 ± 8.7** | **17.0 ± 9.0** | **18.8 ± 8.0** |
|  |  |  |  |  |  |

The comparisons with the pre-menopausal population that were statistically significant with p<0.05 are shown in bold type.

***** Estimated glomerular filtration rate (eGFR) was calculated by chronic kidney disease epidemiology collaboration (CKD-EPI) method.

Abbreviations: AA: African American; AF: atrial fibrillation; Ca: Caucasian; CHD: coronary heart disease; Ch: Chinese American; CVD: cardiovascular disease; DHEA: dehydroepiandrosterone; HDL: high-density lipoprotein; HF: heart failure; Hi: Hispanic; LA: left atrium; LACI: left atrioventricular coupling index; EDVi: end-diastolic volume indexed; ESVi: end-systolic volume indexed; LV: left ventricle; LVEF: left ventricle ejection fraction; LVGFI: left ventricle global function index; LVMVR: LV mass/LV volume; NT-proBNP: N-terminal prohormone of brain natriuretic peptide; SHBG: sex hormone binding globulin; T: testosterone.

**SUPPLEMENT 6:**

**Figure. Distribution of LACI.**

**
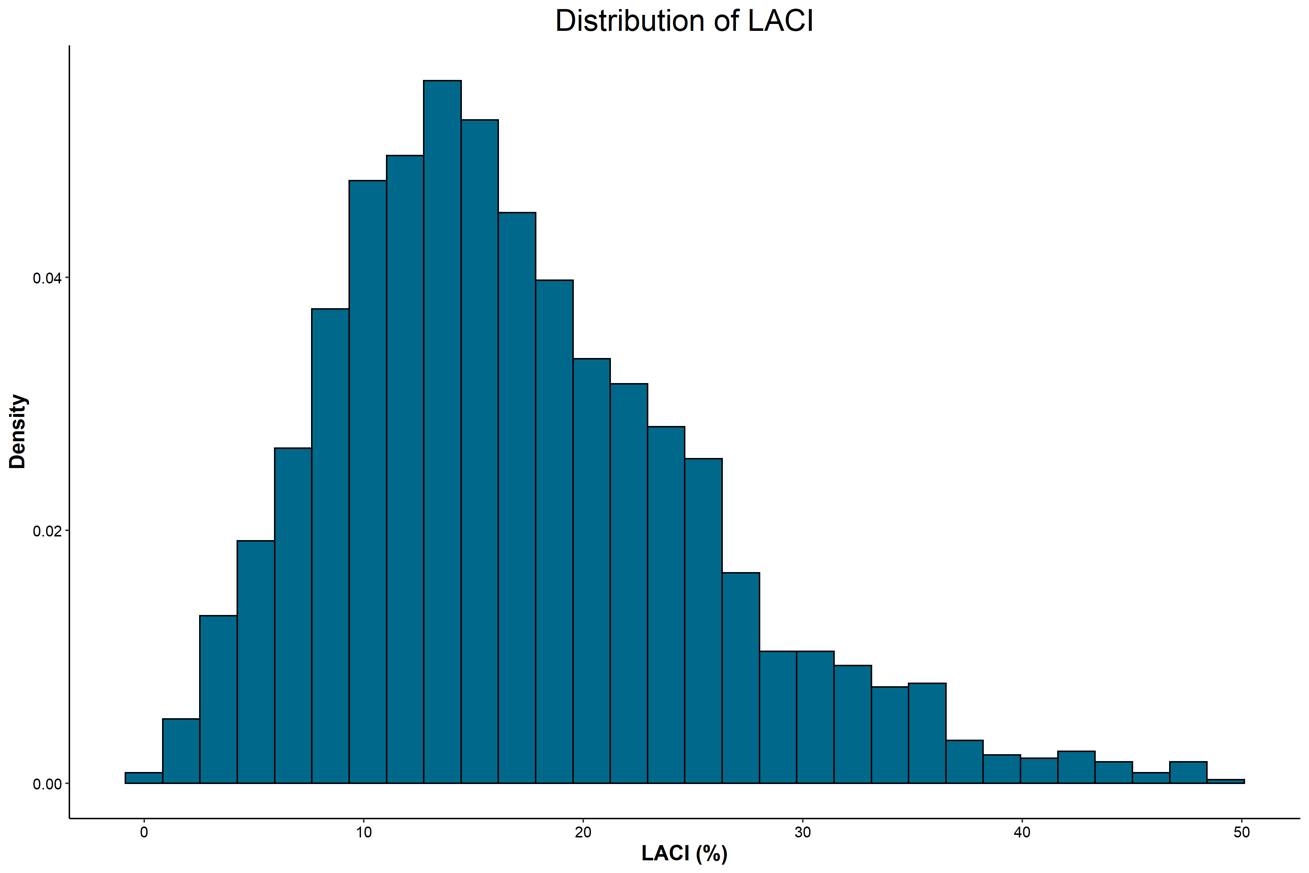
**

**SUPPLEMENT 7:**

**Table. Univariable and multivariable analysis of CV events occurrence according to LACI and other LA/LV parameters or biomarkers and scores.**

| **Outcomes** | **Unadjusted** | | |  | **Adjusted** | | |  |
| --- | --- | --- | --- | --- | --- | --- | --- | --- |
|  | **HR (95%CI)** | **p-value** | **C-index (95%CI)** |  | **HR (95%CI)** | **p-value** | **C-index (95%CI)** |  |
| **Incident AF** |  |  |  |  |  |  |  |  |
| - LACI, % (per 1 SD) | 1.99 (1.73-2.13) | **<0.001** | 0.68 (0.64-0.72) |  | 1.70 (1.51-1.90) | **<0.001** | 0.82 (0.80-0.85) |  |
| - LVGFI, % (per 1 SD) | 0.81 (0.71-0.92) | **0.002** | 0.56 (0.52-0.60) |  | 0.96 (0.84-1.10) | 0.57 | 0.79 (0.77-0.82) |  |
| - LVMVR, g/mL (per 1 SD) | 1.26 (1.11-1.43) | **<0.001** | 0.59 (0.55-0.63) |  | 1.06 (0.92-1.22) | 0.42 | 0.79 (0.77-0.82) |  |
| - LA ESVi, ml/m^2^ (per 1 SD) | 1.44 (1.27-1.62) | **<0.001** | 0.59 (0.55-0.63) |  | 1.35 (1.20-1.52) | **<0.001** | 0.80 (0.78-0.83) |  |
| - CHARGE-AF score, % | 2.57 (2.19-3.02) | **<0.001** | 0.77 (0.75-0.80) |  | 1.45 (0.87-2.40) | 0.15 | 0.79 (0.77-0.82) |  |
| **Incident HF** |  |  |  |  |  |  |  |  |
| - LACI, % (per 1 SD) | 2.05 (1.68-2.37) | **<0.001** | 0.71 (0.65-0.76) |  | 1.62 (1.33-1.97) | **<0.001** | 0.84 (0.80-0.88) |  |
| - LVGFI, % (per 1 SD) | 0.66 (0.56-0.77) | **<0.001** | 0.66 (0.60-0.73) |  | 0.76 (0.64-0.91) | <0.001 | 0.82 (0.80-0.85) |  |
| - LVMVR, g/mL (per 1 SD) | 1.45 (1.22-1.84) | **<0.001** | 0.64 (0.58-0.70) |  | 1.13 (0.92-1.39) | 0.24 | 0.81 (0.77-0.85) |  |
| - LA ESVi, ml/m^2^ (per 1 SD) | 1.57 (1.30-1.89) | **<0.001** | 0.64 (0.58-0.70) |  | 1.40 (1.15-1.70) | **<0.001** | 0.82 (0.80-0.85) |  |
| - Framingham score, % | 2.13 (1.78-2.53) | **<0.001** | 0.73 (0.69-0.78) |  | 0.83 (0.50-1.36) | 0.46 | 0.81 (0.77-0.86) |  |
| **CHD death** |  |  |  |  |  |  |  |  |
| - LACI, % (per 1 SD) | 1.80 (1.49-2.18) | **<0.001** | 0.67 (0.61-0.74) |  | 1.36 (1.10-1.68) | **0.001** | 0.87 (0.84-0.90) |  |
| - LVGFI, % (per 1 SD) | 0.77 (0.64-0.94) | **0.032** | 0.60 (0.52-0.67) |  | 0.82 (0.64-1.04) | 0.10 | 0.85 (0.82-0.88) |  |
| - LVMVR, g/mL (per 1 SD) | 1.56 (1.30-1.88) | **<0.001** | 0.65 (0.58-0.71) |  | 1.18 (0.94-1.46) | 0.15 | 0.85 (0.82-0.89) |  |
| - LA ESVi, ml/m^2^ (per 1 SD) | 1.28 (1.04-1.59) | **0.021** | 0.56 (0.49-0.63) |  | 1.19 (0.97-1.48) | 0.10 | 0.85 (0.82-0.88) |  |
| - Log(Agatston score) | 1.38 (1.20-1.49) | **<0.001** | 0.69 (0.64-0.75) |  | 1.13 (0.46-2.78) | 0.79 | 0.85 (0.82-0.88) |  |
| - Framingham score, % | 1.98 (1.56-2.45) | **<0.001** | 0.79 (0.72-0.83) |  | 1.40 (0.81-2.39) | 0.23 | 0.85 (0.82-0.88) |  |
| **Hard CVD** |  |  |  |  |  |  |  |  |
| - LACI, % (per 1 SD) | 1.65 (1.45-1.88) | **<0.001** | 0.64 (0.60-0.69) |  | 1.30 (1.13-1.51) | **<0.001** | 0.78 (0.74-0.81) |  |
| - LVGFI, % (per 1 SD) | 0.66 (0.57-0.77) | **<0.001** | 0.61 (0.56-0.65) |  | 0.75 (0.64-0.88) | **<0.001** | 0.76 (0.73-0.80) |  |
| - LVMVR, g/mL (per 1 SD) | 1.46 (1.28-1.67) | **<0.001** | 0.62 (0.58-0.67) |  | 1.28 (1.10-1.48) | **0.001** | 0.76 (0.73-0.80) |  |
| - LA ESVi, ml/m^2^ (per 1 SD) | 1.20 (1.03-1.39) | **0.018** | 0.55 (0.50-0.60) |  | 1.12 (0.97-1.30) | 0.13 | 0.76 (0.72-0.79) |  |
| - Log(Agatston score) | 1.31 (1.16-1.45) | **0.003** | 0.66 (0.62-0.72) |  | 1.16 (1.08-1.26) | **0.032** | 0.76 (0.73-0.80) |  |
| - Framingham score, % | 1.86 (1.64-2.12) | **<0.001** | 0.70 (0.68-0.76) |  | 0.96 (0.65-1.41) | 0.83 | 0.76 (0.73-0.80) |  |
|  |  |  |  |  |  |  |  |  |

- All LV parameter, LA parameter and LACI values were normalized according to the following formula: (parameter–mean value)/standard deviation.
- Adjusted model included age, ethnicity, education level, physical activity, menopausal status, hormone therapy (HT) use, diabetes mellitus, current smoking, systolic blood pressure, anti-hypertensive therapy, body mass index, high-density lipoprotein cholesterol, total cholesterol, lipid-lowering therapy, total testosterone/estradiol ratio, DHEA level, and glomerular filtration rate.

Abbreviations: AF: atrial fibrillation; CHD: coronary heart disease; CI: confidence interval; CVD: cardiovascular disease; HF: heart failure; HR: hazard ratio; LA: left atrium; LACI: left atrioventricular coupling index; ESVi: end-systolic volume indexed; LV: left ventricle; LVGFI: left ventricle global function index; LVMVR: LV mass/LV volume ratio.

**SUPPLEMENT 8:**

**Figure. Kaplan-Meier survival curves for incident AF (A), incident HF (B), CHD death (C) and hard CVD (D) stratified by LACI >25%.**

In the overall population, the cumulative hazard was systematically significantly greater for women with LACI >25% compared to women with LACI ≤25% for each outcome (log-rank for difference; p<0.001).


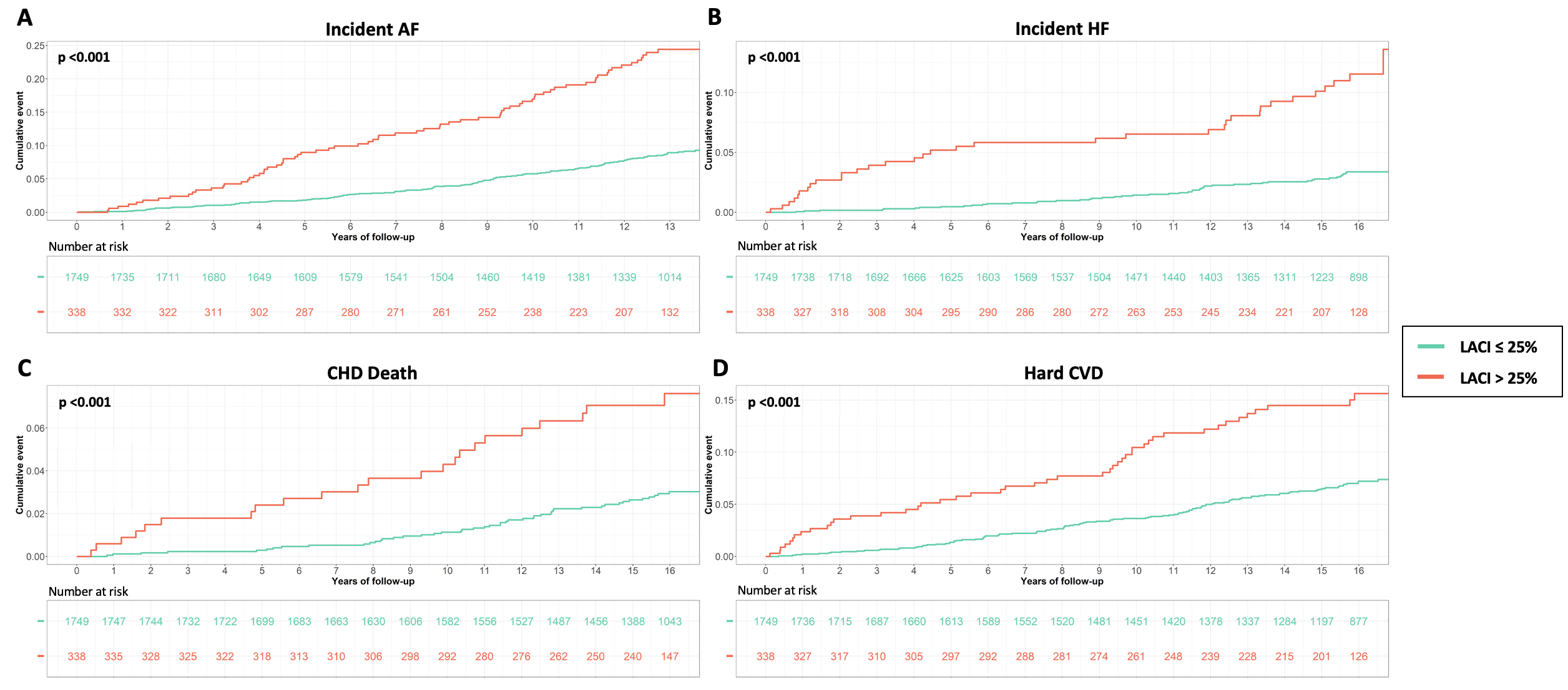


**SUPPLEMENT 9:**

**Figure. Kaplan-Meier survival curves for incident AF (A), incident HF (B), CHD death (C) and hard CVD (D) according to the menopausal status and stratified by LACI >25%.**

The cumulative hazard was systematically significantly greater for post-menopausal women with LACI >25% compared to the other women for each outcome (log-rank for difference; p<0.001).


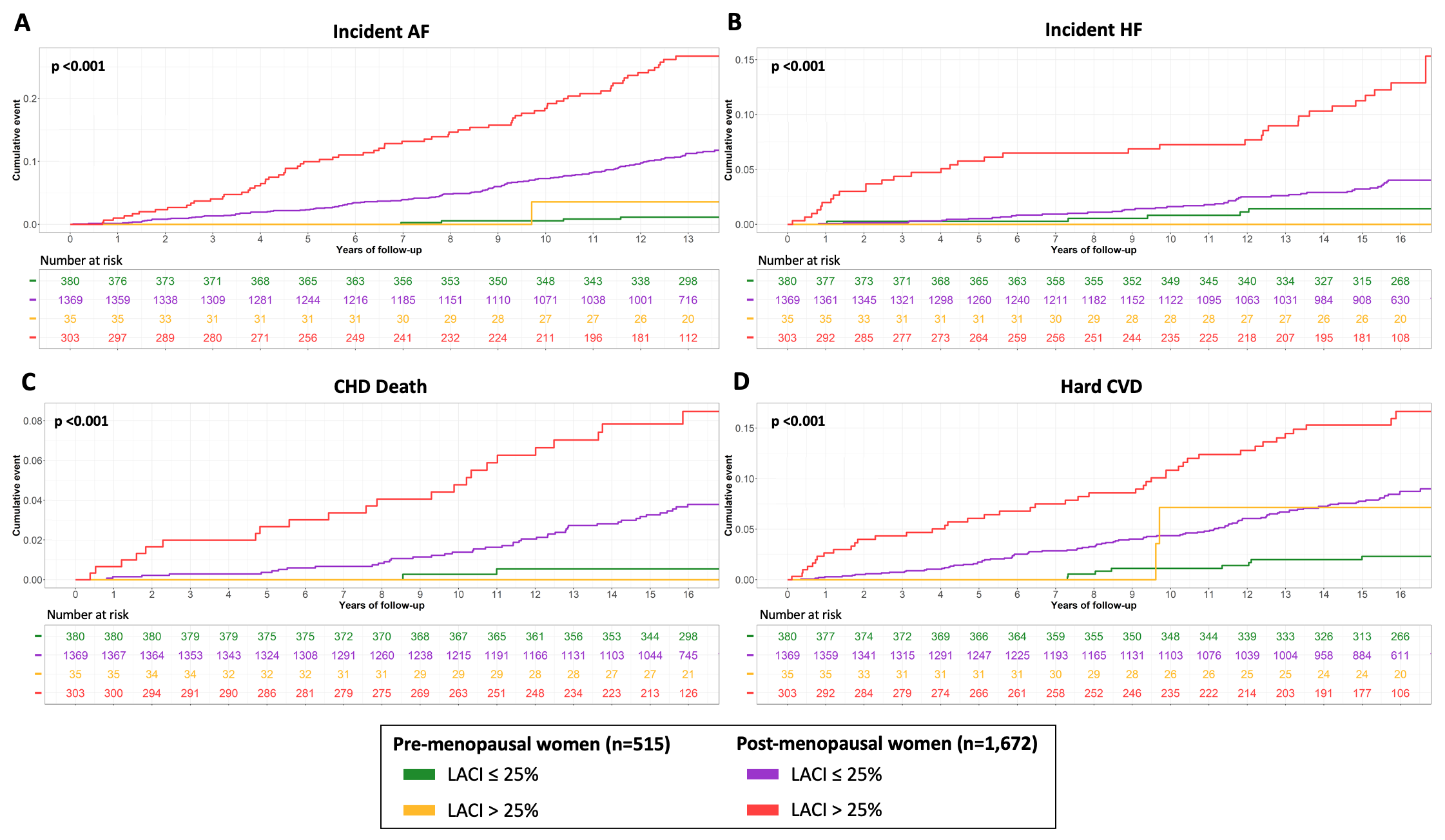


**SUPPLEMENT 10:**

**Table. Discrimination and reclassification associated with LACI to different LA, LV parameters, biomarker and scores (n=2,087).**

| **Model** | **Incident AF** | | **Incident HF** | | **CHD death** | | **Hard CVD** | |
| --- | --- | --- | --- | --- | --- | --- | --- | --- |
|  | **NRI**  (p-value) | **IDI**  (p-value) | **NRI**  (p-value) | **IDI**  (p-value) | **NRI**  (p-value) | **IDI**  (p-value) | **NRI**  (p-value) | **IDI**  (p-value) |
| Multivariable model* | Reference | | Reference | | Reference | | Reference | |
| Multivariable model with LACI^†^ | 0.325  (**<0.001**) | 0.036  (**<0.001**) | 0.571  (**<0.001**) | 0.023  (**<0.001**) | 0.506  (0.06) | 0.012  (**0.010**) | 0.229  (**0.010**) | 0.012  (**<0.001**) |
| Multivariable model with LA EDVi | 0.251  (**0.002**) | 0.025  (**<0.001**) | 0.467  (**0.040**) | 0.22  (**0.002**) | 0.271  (0.15) | 0.003  (0.23) | 0.239  (0.16) | 0.004  (0.61) |
| Multivariable model with LV EDVi | 0.139  (0.52) | 0.002  (0.31) | 0.039  (0.23) | 0.009  (0.08) | 0.568  (0.95) | 0.000  (0.85) | 0.285  (0.17) | 0.009  (0.11) |
| Multivariable model with LVEF | 0.098  (0.47) | -0.001  (0.88) | 0.339  (**0.008**) | 0.014  (**0.040**) | 0.126  (0.57) | -0.001  (0.86) | 0.143  (0.21) | 0.002  (0.32) |
| Multivariable model with LVGFI | -0.003  (0.38) | -0.000  (0.55) | 0.356  (**<0.001**) | 0.012  (**0.042**) | 0.375  (0.37) | 0.001  (0.53) | 0.219  (**0.010**) | 0.009  (**0.02**) |
| Multivariable model with LV mass | 0.076  (0.65) | 0.001  (0.50) | 0.211  (**0.002**) | 0.012  (**0.041**) | -0.252  (0.74) | -0.001  (0.98) | -0.052  (0.79) | -0.001  (0.98) |
| Multivariable model with LVMVR | 0.027  (0.60) | 0.001  (0.34) | 0.096  (0.53) | 0.002  (0.28) | 0.263  (0.37) | 0.005  (0.08) | 0.223  (0.06) | 0.011  (**<0.001**) |
| Multivariable model with Peak LA strain | 0.293  (**<0.001**) | 0.014  (**<0.001**) | 0.506  (**0.001**) | 0.009  (**<0.001**) | 0.5322  (0.90) | 0.001  (0.87) | 0.103  (0.62) | 0.000  (0.48) |
| Multivariable model with LA ESVi | 0.200  (0.070) | 0.011  (**0.020**) | 0.191  (0.20) | 0.008  (0.16) | 0.300  (0.22) | -0.000  (0.77) | 0.164  (0.46) | -0.002  (0.59) |
| Multivariable model with Log (Agatston score) | - | - | - | - | 0.376  (0.34) | 0.005  (0.23) | 0.498  (0.25) | 0.001  (0.65) |
| Multivariable model with Framingham score | - | - | -0.003  (0.52) | 0.001  (0.47) | 0.461  (0.17) | 0.003  (0.19) | 0.095  (0.72) | 0.001  (0.79) |
| Multivariable model with CHARGE-AF score | -0.010  (0.91) | -0.002  (0.40) | - | - | - | - | - | - |

- All LV parameter, LA parameter and LACI values were normalized according to the following formula: (parameter–mean value)/standard deviation.
- For each model, discrimination and reclassification were based on net reclassification improvement (NRI) and integrated discrimination improvement (IDI). Results are for 10-year follow-up.

* Multivariable model included age, ethnicity, education level, physical activity, menopausal status, hormone therapy (HT) use, diabetes mellitus, current smoking, systolic blood pressure, anti-hypertensive therapy, body mass index, high-density lipoprotein cholesterol, total cholesterol, lipid-lowering therapy, total testosterone/estradiol ratio, DHEA level, and glomerular filtration rate.

† LACI used as continuous variable

Abbreviations: AF: atrial fibrillation; CHD: coronary heart disease; CVD: cardiovascular disease; HF: heart failure; HR: hazard ratio; IDI: integrative discrimination index LA: left atrium; LACI: left atrioventricular coupling index; EDVi: end-diastolic volume indexed; ESVi: end-systolic volume indexed; LV: left ventricle; LVEF: left ventricle ejection fraction; LVGFI: left ventricle global function index; LVMVR: LV mass/LV volume; NRI: net reclassification improvement.
